# Validation of a Nutrition Screening Tool in Critically Ill Children

**DOI:** 10.3390/nu18142340

**Published:** 2026-07-16

**Authors:** Anna Burneske, Collin Ellenbecker, Evelyn Kuhn, Jacob Swoveland, Matt Oelstrom, Scott Hagen, Sarah Mandli, Lynne Sears, Jennifer Peterson, Charlene P. Pringle, Kelly Sheridan, Megan Foxe, Abigail Hebron, Elizabeth Zivick, Nicole Fabus, Rebecca Heisler, Melissa Froh, Sadaf Shad, Theresa Mikhailov

**Affiliations:** 1 Division of Critical Care, Department of Pediatrics, Medical College of Wisconsin, Milwaukee, WI 53226, USA; 2 Division of Critical Care, Department of Pediatrics, University of Wisconsin, Madison, WI 53792, USA; 3 Children’s Hospital of Wisconsin, Milwaukee, WI 53226, USA; 4 Marshfield Clinic, Marshfield, WI 54449, USA; 5 Gundersen Health Systems Hospitals and Clinics, La Crosse, WI 54601, USA; 6 Division of Critical Care, Department of Pediatrics, University of Florida, Gainesville, FL 32610, USA; 7 Division of Critical Care, Department of Pediatrics, Medical University of South Carolina, Charleston, SC 29425, USA

**Keywords:** malnutrition, nutrition screening tool, critically ill child, enteral nutrition, nutritional support, pediatric nutrition, clinical nutrition

## Abstract

**Background/Objectives:** Malnutrition is prevalent among patients in the pediatric intensive care unit (PICU) and has been shown to worsen in some patients during the PICU stay. In critically ill children, malnutrition is associated with longer PICU length of stay and increased mortality. Several tools have been developed and validated to screen for nutritional status in children, but none were specifically designed for critically ill children. Our study aims to fill this gap. The goal of this study was to refine and validate a novel PICU nutrition screening tool in a diverse population of critically ill children from six PICUs across the United States. **Methods:** Subjects underwent the nutrition screen and a Subjective Global Nutritional Assessment (SGNA). We used chi-square tests and Mann–Whitney tests to compare elements of the nutrition screen to the SGNA to identify those elements most associated with malnutrition. We used stepwise logistic regression to determine the best fitting model for a malnutrition screening tool. We proposed a scoring system using the factors identified in the best fitting model to define a positive screen. **Results**: We enrolled 732 subjects at six PICUs. Of these, 131 subjects (17.9%) were malnourished per the SGNA. Children with and without malnutrition did not differ with respect to age, sex, or race. The likelihood of malnutrition increased as weight-for-age percentile decreased (*p* < 0.001). We found that the best fitting model of a malnutrition screening tool for critically ill children included weight-for-age percentile, cancer, feeding less, parent perception of growth as poor, and being significantly underweight. We defined a positive screen that should prompt referral to a dietitian. **Conclusions:** We have developed a nutrition screening tool for critically ill children.

## 1. Introduction

Malnutrition is prevalent among patients in the pediatric intensive care unit (PICU) and has been shown to worsen in some patients during the PICU stay [1]. In critically ill children, malnutrition is associated with longer PICU length of stay and increased mortality. Previous work has shown that 24–53% of critically ill or injured children are malnourished at the time of admission [2,3,4]. Using a nutrition screening tool designed for this patient population would allow early identification of patients at risk for malnutrition. This would then allow for prompt and appropriate interventions to optimize their clinical course.

PICU patients are often undergoing more interventions than their acutely ill counterparts as a result of the severity of their illness. They may require fluid resuscitation or may be fluid-restricted, which can result in significantly altered intake. Energy requirements may increase during critical illness and the profound inflammatory response in critical illness may affect nutrition status [5,6]. An additional clinical workflow hurdle to nutrition screening in the PICU is the high number of tasks that must be completed rapidly at the time of admission to the PICU. The ideal screening tool would be easy and quick to accomplish in order to be performed during the busy start of an admission, during which nursing and other allied health care staff are responsible for many other equally important tasks. Timing is more challenging due to the PICU workflow.

The benefits of early enteral nutrition are well recognized; however, early intervention requires identification through a screening process [7,8,9]. Several tools have been developed and validated to screen for nutritional status in children, but none were specifically designed or validated for critically ill children [10,11,12,13]. Our study aims to fill this gap by developing and validating a nutrition screening tool designed specifically for the PICU population.

In Phase I of this study, we developed a preliminary nutrition screening tool at the Children’s Hospital of Wisconsin using data from 394 PICU patients. In Phase II, we validated the tool in a larger population from six PICUs across the United States by comparing it to the Subjective Global Nutritional Assessment (SGNA), which has been validated in critically ill children. The goal of Phase II was to refine and validate the screening elements that most accurately indicated malnutrition. 

## 2. Methods

We obtained IRB approval for both Phase I and Phase II from the Children’s Hospital of Wisconsin. All sites participating in Phase II obtained IRB approval at their respective institutions.

In the Phase I study, we evaluated 30 clinical factors and anthropometric measures found in nutrition screening tools designed for acutely ill children to determine which factors were most closely associated with malnutrition in critically ill children. A member of the research team completed this nutrition screen for 437 patients, and a registered dietitian (RD) completed the SGNA for 394 of those patients within 24 h of admission to the hospital. We were unable to complete the SGNA on the remaining 43 patients for various reasons (e.g., the patient was unavailable, the parent was unavailable, or the patient had been transferred out of the PICU). We compared the 30 clinical factors and anthropometric measures to the SGNA using univariate analysis and multiple logistic regression to determine which factors were the most accurate indicators of malnutrition. Based on this analysis, we reduced the list of factors to nine, which we then considered to be the preliminary nutrition screen for Phase II. We included these nine variables in our novel screen for Phase II and obtained age, weight, and height at admission for each subject. We determined weight-for-age percentile and height-for-age percentile for all subjects. All anthropometric data were obtained at admission or shortly thereafter and all screens and SGNAs were completed within 24 h of admission.

In Phase II, 760 subjects underwent the preliminary nutrition screen and 732 of these underwent the SGNA. The SGNA was not completed on the remaining 28 patients for the same reasons as in Phase I. We included all patients who were at least 30 days old and less than 18 years old. We excluded non-English-speaking patients and children who had already participated in this research study during a previous admission to the PICU.

We used chi-square tests and Mann–Whitney tests as appropriate to compare elements of the preliminary nutrition screen to the SGNA determination of nutritional status to identify those elements most strongly associated with malnutrition. We used logistic regression analysis to determine the best fitting model of a malnutrition screening tool for critically ill children. We assigned one point for each affirmative response for the four dichotomous selected criteria and one point for weight-for-age percentile ≤ 1 standard deviation below the mean. We calculated accuracy, sensitivity, specificity, positive predictive value, and negative predictive value based on the data available in our subjects. One participating site lost dietitian coverage for the research activities in their PICU and trained a senior medical student to perform the SGNA for the remaining patients in the study. We conducted a sensitivity analysis to determine the impact of this personnel change on data integrity.

The sample size for Phase I was based on a desired sensitivity of 90% with a target confidence interval of 15% and an estimated proportion of malnourished subjects (i.e., 20%) in the PICU. This yielded a sample size of 370 for Phase I. The sample size for Phase II was determined by the number of sites participating, the proportion of malnourished subjects (i.e., 20%) in the PICU, and a desired sensitivity of 90% with a target confidence interval of +/−5%. This yielded an estimated sample size between 600 and 1200 subjects. The high sensitivity was used in both Phase I and Phase II to minimize false negative screens.

**Figure 1:**
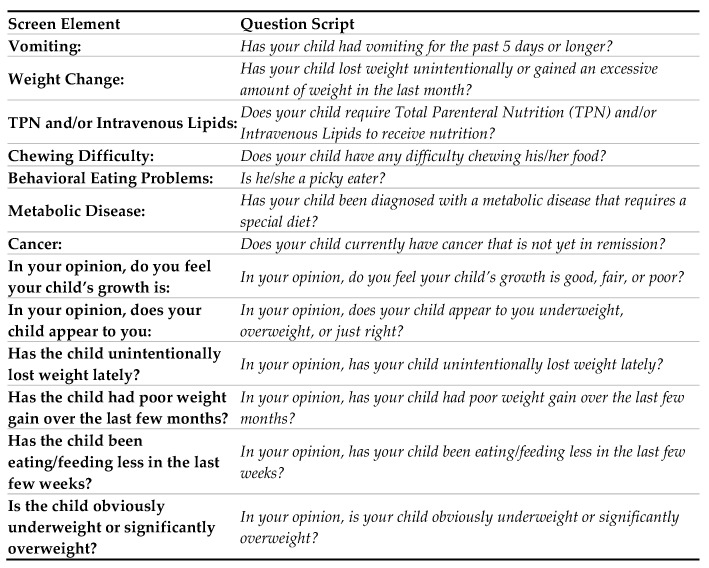
Standardized question script for each screen element.

The research team at the lead site created a Manual of Operations (MOO) that outlined the study protocol, the training plan for research staff and dietitians, the screening process, the informed consent process, and the data collection process. The MOO included a script for obtaining informed consent, a script for conducting the nutrition screen in consented subjects (see Figure 1), and detailed directions for performing the SGNA. The research team at the lead site also created a standard presentation for training dietitians on how to perform the SGNA. This training was conducted virtually using a commercially available software program. This training included a power point presentation with step-by-step demonstration of the SGNA. This was led by a research coordinator and the lead dietitian from the lead site. These training sessions allowed an interactive experience for dietitians at each site to learn how to conduct the SGNA. The lead dietitian was also available for questions from dietitians at participating sites on an as-needed basis.

In both study phases, we calculated IRR (inter-rater reliability) for the SGNAs performed by participating dietitians using intraclass correlations. For Phase I IRR testing, we considered the most experienced dietitian’s rating (i.e., Rating 1) to be the final answer. For Phase II IRR testing, we calculated intraclass correlations between eight individual raters. We calculated this for all three categories of response (i.e., normal, moderate, and severe). Due to the low numbers of patients categorized as severe, we also calculated the intraclass correlation for two categories of response by combining moderate and severe into a single category of abnormal.

## 3. Results

Based on Phase I analysis, the following variables were considered appropriate for further consideration: vomiting, weight change, TPN/IL, cancer diagnosis, parent’s perception that child is underweight, parent’s perception of child’s growth, chewing difficulty, behavioral problems with eating, and presence of a metabolic disorder. We also included the Pediatric Nutrition Screening Tool (PNST) questions in our data collection to allow us to compare our novel nutrition screen to the PNST.

For Phase II, we prospectively enrolled 760 subjects at six PICUs and completed both the preliminary nutrition screen and the SGNA on 732 subjects. Of the 732 subjects in Phase 2, 131 (17.9%) were malnourished (121 were moderately malnourished, and 10 were severely malnourished), as determined by the SGNA. The children with malnutrition did not differ from the children without malnutrition with respect to age, sex, or race (see Table 1). Several elements of the preliminary nutrition screen were associated with malnutrition (Table 2). The likelihood of malnutrition increased as weight-for-age percentile decreased (*p* < 0.001). The best fitting model of a malnutrition screening tool for critically ill children included weight-for-age percentile and four elements of the preliminary nutrition screening tool (cancer diagnosis, eating less, obviously underweight, and poor growth) (Table 3). The sensitivity analysis excluded all patients whose SGNA was performed by the non-dietitian and determined that there was no appreciable impact on the best fitting model.

Based on the distribution of weight-for-age percentiles in the patients who were malnourished on the SGNA compared to that of the patients who were not malnourished on the SGNA, a cutoff of one standard deviation below the mean, (i.e., the 16th percentile) was considered to be an affirmative response. Also, one point was assigned for each affirmative response for the remaining elements (cancer diagnosis, eating less, obviously underweight, and poor growth). Based on this methodology, we reviewed the tradeoff between sensitivity and specificity to determine a proposed screen. A cutoff of at least 2 points yielded the highest accuracy with acceptable sensitivity and specificity as well as positive and negative predictive values (see Table 4). Thus, 2 points constituted a positive screen. 

In Phase I, we performed the IRR on 34 (7.8%) of our subjects and found agreement on 31 of the 34 subjects (*p* = 1.00). In Phase II, the IRR based on an intraclass correlation coefficient (ICC) and three categories for eight raters was 0.653 (95%CI 0.551–0.752). When the moderate category was combined with severe, the IRR was 0.656 (95%CI 0.556–0.755).

## 4. Discussion

We have developed and validated a nutrition screening tool for critically ill children that incorporates weight-for-age percentile and four additional elements that can be obtained through questions asked of the parent or guardian at admission to the PICU. These questions can be asked by any staff member and do not require an RD or other specially trained provider. 

This project evaluates specific factors previously presumed to be clinically relevant in a large population of acutely ill children to determine which are the best predictors of nutrition status deterioration in critically ill children. There are limited studies which do this. Furthermore, these questions can be asked by any staff member and do not require an RD or other specially trained provider. The major nutrition screening tools were designed for acutely ill children and have not been validated in the critically ill patient population. While many studies have evaluated their performance in acutely ill children, this work is valuable in that it evaluates the components assessed in many of these tools specifically in a relatively large PICU population and compares them to a gold standard assessment of nutritional status. 

Our findings are similar to those in the 1997 study which validated a nutrition screening tool in pediatric patients in medical and surgical wards [14]. It found decreased oral intake (defined as food intake < 50%) to be a significant nutritional risk factor. This is comparable to what we found with the variable “eating or feeding less.” However, our findings differ in that Sermet-Gaudelus did not examine the caregiver’s opinion or any anthropometric measures such as weight-for-age percentile. A large 2016 study by Chourdakis found that rates of nutritional support (defined to include oral supplements, tube feeding, or parenteral nutrition) were higher among patients identified by the Pediatric Yorkhill Malnutrition Score (PYMS) to be at high risk of malnutrition than those who were at medium or low risk [15]. In our study, although TPN was associated with risk of malnutrition on univariate analysis, the association was not strong enough to remain associated with risk of malnutrition in the multivariate analysis and therefore is not an element of our nutrition screen. Our study is more recent and was conducted exclusively in PICU patients within the US rather than Europe. Thus, differences may reflect changes in clinical practices over time as well as the difference in patient populations. 

Hulst et al. found energy and protein deficits were associated with lower SD-scores for weight and arm circumference in patients in the PICU [1]. While we did not study energy and protein deficits, it stands to reason that these might be found in patients who have been “eating or feeding less” or who are considered by their caregiver to have lost weight. The Strong Kids found a significant relationship between having a ‘‘high risk’’ Strong Kids score and a negative SD-score in weight-for-height [13]. While this study was not performed in critically ill children, it does suggest anthropometric measurements are a critical component when screening for malnutrition. 

The Children’s Wisconsin Nutrition Screening Tool (CWNST), which is a combination of the PNST and predictive elements from the electronic medical record, included parenteral nutrition, positive PNST, and BMI-for-age or weight-for-length z-score. While our work examined weight-for-age vs BMI-for-age/weight-for-length z-score, this is a comparable finding in that it deals with anthropometric parameters as a possible indicator of malnutrition. Components of the PNST are included in our screen: whether or not the child has been eating or feeding less and whether or not the child is obviously underweight. It is also worth noting that the next best fitting model was PNST + weight-for-age percentile.

When the SGNA is compared strictly to anthropometric measurements in the PICU, there is a strong and significant correlation with its determination of nutrition status and anthropometric measurements [16]. Below-normal anthropometric measures may be associated with malnutrition [17]. This is consistent with what we have shown in this work, as weight-for-age percentile was found to be statistically significant. 

Aspects of this project were limited by the COVID 19 pandemic. The number of subjects enrolled was limited due to the reassignment of RD workload from research to other clinical areas. Patient census and type of patient acuity/were impacted by the pandemic as well. No site was able to approach every potential eligible patient due to staffing limitations (weekends, holidays). Another important limitation of this study is the exclusion of non-English-speaking subjects. The inclusion of non-English-speaking subjects would have required translation of consent documents into Spanish and possibly several other languages by a certified translation provider at each site and funding for this service was not available.

Future work should include a prospective study with the newly proposed nutrition screening tool described here (the best fit model and the CWNST) applied to patients in other institutions’ PICUs. This nutrition screen and standard RD assessment would be performed on all eligible admitted patients. The results could also be compared to the SGNA. Analysis to determine the sensitivity and specificity of these tools would be beneficial as well. 

## 5. Conclusions

We have developed and validated a nutrition screening tool for critically ill children that incorporates weight-for-age percentile and four additional elements that can be obtained through questions asked of the parent or guardian at admission to the PICU. We assigned 1 point for each affirmative response and 1 point for weight-for-age percentile ≤ 16th percentile score. For this model, a score of at least 2 points is a positive screen and indicates a need for prompt evaluation by a dietitian.

## Figures and Tables

**Table 1:** Demographics.

**Age (Years)**		
	<2 years	341 (46.58)
	2 years to <6 years	168 (22.95)
	6 years to <12 years	101 (13.79)
	12 years to <18 years	122 (16.67)
**Gender**	Female	353 (48.22)
	Male	378 (51.63)
**Race**	American Indian/Alaska Native	4 (0.55)
	Asian	12 (1.64)
	Black/African American	155 (21.17)
	Native Hawaiian or Other Pacific Islander	1 (0.14)
	Patient Refused	9 (1.23)
	White	516 (70.50)
	Missing	35 (4.00)
**SGNA**	Malnourished	131 (17.89)
	Not Malnourished	601 (82.10)

Legend: Data presented as n (%).

**Table 2:** Univariate comparison of preliminary nutrition screening tool elements and SGNA determination of nutritional status.

Screen Element	Positive SGNA N (%)	Negative SGNA N (%)	Odds Ratio (95% CI)	*p*-Value
**Vomiting:**				0.0035
**Yes**	63 (23.5%)	205 (76.5%)	1.78 (1.19–2.67)	
**No**	66 (14.7%)	383 (85.3%)		
**Weight Change:**				<0.001
**Yes**	50 (35.2%)	92 (64.8%)	3.4 (2.18–5.27)	
**No**	79 (13.8%)	495 (86.2%)		
**TPN and/or Intravenous Lipids:**				0.044
**Yes**	8 (36.4%)	14 (63.6%)	2.64 (0.93–6.95)	
**No**	105 (17.8%)	486 (82.2%)		
**Chewing Difficulty:**				0.044
**Yes**	16 (27.1%)	43 (72.9%)	1.93 (0.96–3.71)	
**No**	77 (16.1%)	400 (83.9%)		
**Behavioral Eating Problems:**				0.27
**Yes**	39 (21.0%)	147 (79.0%)	1.29 (0.82–1.99)	
**No**	90 (17.1%)	437 (82.9%)		
**Metabolic Disease:**				0.32
**Yes**	7 (25.0%)	21 (75.0%)	1.55 (0.54–3.89)	
**No**	122 (17.7%)	567 (82.3%)		
**Cancer:**				0.0024
**Yes**	10 (45.5%)	12 (54.5%)	4.02 (1.52–10.41)	
**No**	119 (17.1%)	575 (82.9%)		
**In your opinion, do you feel your child’s growth is:**				<0.001
**Good**	68 (12.2%)	490 (87.8%)	Chi-square	
**Fair**	34 (29.6%)	81 (70.4%)	3 *df*	
**Poor**	25 (69.4%)	11 (30.6%)	88.045	
**Unsure**	2 (25.0%)	6 (75.0%)		
In your opinion, does your child appear underweight to you? *				<0.001
**Yes**	50 (41.7%)	70 (58.3%)	4.67 (2.95–7.37)	
**No**	79 (13.2%)	518 (86.8%)		
**Has the child unintentionally lost weight lately?**				<0.001
**Yes**	40 (45.5%)	48 (54.5%)	5.069 (3.056–8.39)	
**No**	88 (14.1%)	537 (85.9%)		
**Has the child had poor weight gain over the last few months?**				<0.001
**Yes**	25 (69.4%)	11 (30.6%)	12.54 (5.74–29.14)	
**No**	104 (15.3%)	577 (84.7%)		
**Has the child been eating/feeding less in the last few weeks?**				<0.001
**Yes**	69 (35.8%)	124 (64.2%)	4.27 (2.82–6.51)	
**No**	60 (11.5%0	462 (88.5%)		
**Is the child obviously underweight?**				<0.001
**Yes**	37 (46.8%)	42 (53.2%)	5.16 (3.05–8.72)	
**No**	92 (14.5%)	541 (85.5%)		

Legend: For each screen element, all study personnel used the script as written to maintain uniformity across sites. Data were compared by chi-square test of proportions of each screen element to SGNA. * The *p*-value for this screen element was based on response of “underweight” rather than “overweight” or “just right”.

**Table 3:** Best fitting model for a nutrition screening tool for critically ill children based on multivariate logistic regression analysis.

Screen Element	Odds Ratio	95% Confidence Interval	*p*-Value
**Cancer**	4.67	1.64–13.15	0.003
**Has child been eating/feeding less in the last few weeks?**	4.22	2.68–6.69	<0.001
**Is child obviously underweight?**	2.36	1.26–4.36	0.007
**Parent/guardian’s opinion of the child’s growth is poor**	4.66	2.01–11.36	<0.001
**Weight-for-age percentile**	0.98	0.97–0.98	<0.001

Legend: Screen elements listed constitute the best fitting model for the determination of malnutrition based on the gold standard SGNA. Area Under the ROC Curve = 0.808 (95% CI: 0.765–0.851). The weight-for-age percentile factor indicates that, for each 1 percentile increase in weight-for-age, the risk of malnutrition decreased by 0.02.

**Table 4:** Sensitivity, specificity, accuracy, and predictive values of proposed nutrition screen.

	Cut Off: 1+ Points	Cut Off: 2+ Points	Cut Off: 3+ Points
Sensitivity	0.8217	0.4961	0.2248
Specificity	0.5680	0.9133	0.9864
Accuracy	0.6137	0.8382	0.8494
Positive Predictive Value	0.2944	0.5565	0.7838
Negative Predictive Value	0.9356	0.8920	0.8529

## Data Availability

The original contributions presented in this study are included in the article. Further inquiries can be directed to the corresponding author.
